# Static Analysis of Large-Scale Multibody System Using Joint Coordinates and Spatial Algebra Operator

**DOI:** 10.1155/2014/409402

**Published:** 2014-06-19

**Authors:** Mohamed A. Omar

**Affiliations:** Mechanical Engineering Department, Taibah University, Almadinah Almonawwarah 42353, Saudi Arabia

## Abstract

Initial transient oscillations inhibited in the dynamic simulations responses of multibody systems can lead to inaccurate results, unrealistic load prediction, or simulation failure. These transients could result from incompatible initial conditions, initial constraints violation, and inadequate kinematic assembly. Performing static equilibrium analysis before the dynamic simulation can eliminate these transients and lead to stable simulation. Most exiting multibody formulations determine the static equilibrium position by minimizing the system potential energy. This paper presents a new general purpose approach for solving the static equilibrium in large-scale articulated multibody. The proposed approach introduces an energy drainage mechanism based on Baumgarte constraint stabilization approach to determine the static equilibrium position. The spatial algebra operator is used to express the kinematic and dynamic equations of the closed-loop multibody system. The proposed multibody system formulation utilizes the joint coordinates and modal elastic coordinates as the system generalized coordinates. The recursive nonlinear equations of motion are formulated using the Cartesian coordinates and the joint coordinates to form an augmented set of differential algebraic equations. Then system connectivity matrix is derived from the system topological relations and used to project the Cartesian quantities into the joint subspace leading to minimum set of differential equations.

## 1. Introduction

Virtual development procedures became the most economical venue in product design and optimization including earth-moving equipment and automotive systems. These systems are typically large and complex and composed of heterogeneous physical subsystems. Simulation of such systems may require multidomain modeling, for example, rigid and flexible bodies, nonlinear contact and friction force modules, terrain interaction, electrical and hydraulic subsystems, control systems, and nonholonomic constraints. Accurate multibody solver will be required in the heart of the analysis of such systems. As per Giampiero and Manfred [1], the computation of static equilibrium is often the first step in the analysis of multibody system models. The static equilibrium position defines the working point for the linearization of the system equation of motion and provides initial values for the dynamical simulation.

Large earth-moving equipment, agricultural machinery, tracked vehicles, tractors, bulldozers, motor graders, wheeled loaders, and forestry machines are some of the equipment that may work on slopped terrain. Stability assessment of such machinery on the slopes under different loading conditions and different articulation configurations requires accurate modeling of the machine and its interaction with the terrain. The behavior of existing terrain models, in most cases [2–5], is highly nonlinear and history dependent. Performing static equilibrium is very crucial step for accurate linear analysis and stability assessment of the multibody systems.

Another example would be when the multibody dynamics approach is used to model and simulate the startup and shut down of reciprocating engines and turbines which are supported by journal bearings. Journal bearings are typically nonlinear components and their dynamic characteristics (stiffness, damping, etc.) are history dependent [6, 7]. In such systems, the crankshaft and the rotor could be modeled as flexible (deformable bodies). The static equilibrium position of rotor center with respect to the bearing geometric center needs to be accurately determined in order to perform transient simulation or normal mode analysis. When the multibody system contains contacting bodies that may undergo slipping, static equilibrium cannot be obtained from force balance. In this case, the traditional static equilibrium solution will not converge to the true static equilibrium position.

In general, analysis and simulation scenarios of dynamic multibody systems could be summarized as follows:

1. performing transient dynamic simulation of typical machine working cycle or operation;
2. performing eigenvalue analysis to calculate the natural frequencies and normal modes and assess the system stability;
3. determining the static equilibrium of an articulated system that may include rigid and flexible bodies connected with joints.

Three traditional approaches have been used to formulate the multibody system equations of motion: the Cartesian body coordinates (absolute coordinates), the joint coordinates, and velocity transformation method. The Cartesian body coordinates formulations are very popular and reported to be a simpler method to construct the equations of motion leading to a large set of differential algebraic equations [8, 9]. The configuration of a rigid body is described by a set of translational and rotational coordinates. Algebraic constraints are introduced to represent kinematic joints connecting bodies and then the Lagrange multiplier technique is used to describe joint reaction forces. The system of differential algebraic equations has to be solved simultaneously. Newton-Raphson iterations or similar techniques could be used to satisfy the applied constraints. Kinematic assembly of the system is necessary for starting a successful simulation.

Featherstone [10, 11] used spatial vectors to study the dynamics of articulated bodies. Featherstone and Orin [12] and Featherstone [13] presented an efficient approach for utilizing spatial algebra to model multibody systems and efficiently factor the inertia matrix for rigid body systems. Wehage and Haug [14] used a similar approach to develop set of automated procedures for robust and efficient solution of overconstrained multibody dynamics. Wehage and Belczynski [15] proposed structuring the kinematic and dynamic equations into block matrix structure and developed procedures to enable real-time simulation of multibody system. Rodriguez et al. [16, 17] presented a spatial operator based on the spatial algebra for developing multibody dynamic equations of motion. Determining the static equilibrium position using these formulations was not explicitly explained.

Traditional approach of solving for the static equilibrium is based on minimizing the potential energy of the system [1, 18]. This approach requires explicit expression for potential energy of all the bodies and the force elements in the system. This could be practically impossible for the abovementioned heterogeneous system simulations. Other approaches [19, 20] assume the system will be under static equilibrium when the first and second derivatives of the system generalized coordinates are set to zero. The differential equations of motion of the system then become a set of algebraic equations that could be solved along with the constraint equations. The resulting solution yields a set of initial values for the system states. Linear modes analysis could then be performed by carrying out Taylor expansion while only the linear terms are retained [1, 19]. In the abovementioned approaches, false equilibrium position could be reported. Also, violation of the constraints in the velocity level and acceleration level may exist.

Recently, Yang et al. [19] presented a general approach for solving the eigenvalue problem after calculating static equilibrium. The approach is similar to many existing solution approaches and could be summarized by [19] as follows. The system equations of motion could be derived from

(1) $$\begin{array}{c} \frac{d}{dt}\left(\frac{\partial L}{\partial \dot{x}}\right)+\left(\frac{\partial L}{\partial x}\right)={\left(\frac{\partial \Phi }{\partial x}\right)}^{T}\lambda +f,\\ \Phi \left(x\right)=0, \end{array}$$

where **x** ∈ *R* ^*n*^ is the vector of general coordinates, ***λ*** ∈ *R* ^*m*^ is the vector of Lagrange multipliers, *L* is the Lagrange function associated with $\left(x,\;\dot{x}\right)$, **f** is the vector of nonconservative forces which are function of $\left(x,\;\dot{x,}\;t\right)$, Φ is the vector of constraints associated with (**x**), and (∂Φ/∂**x**)^*T*^ ***λ*** is the vector of generalized constraint forces.

The general approach to solve for the static equilibrium position starts by writing the objective function as follows:

(2) $$\begin{array}{c} F\left(\ddot{x},\dot{x},x,t\right)=\left(-\frac{d}{dt}\left(\frac{\partial L}{\partial \dot{x}}\right)+\frac{\partial L}{\partial x}+f\right)\left(\ddot{x},\dot{x},x,t\right),\\ G\left(x\right)={\left(\frac{\partial \Phi \left(x\right)}{\partial x}\right)}^{T}. \end{array}$$

Then, the system equations could be written as

(3) $$\begin{array}{c} F\left(\ddot{x},\dot{x},x,t\right)+G\left(x\right)\lambda =0,\\ \Phi \left(x\right)=0. \end{array}$$

The equilibrium state (**x**_0_, ***λ***_0_, *t*_0_) satisfies the following conditions:

(4) $$\begin{array}{c} F\left(0,0,x_{0},t_{0}\right)+G\left(x_{0}\right)\lambda_{0}=0,\\ \Phi \left(x_{0}\right)=0. \end{array}$$

With a small vibration about the equilibrium position $\left(\delta \ddot{x},\delta \dot{x},x_{0}+\delta x,\lambda_{0}+\delta \lambda ,t\right)$, then

(5) $$\begin{array}{c} F\left(\delta \ddot{x},\delta \dot{x},x_{0}+\delta x,t\right)+G\left(x_{0}+\delta x\right)\left(\lambda_{0}+\delta \lambda \right)=0,\\ \Phi \left(x_{0}+\delta x\right)=0. \end{array}$$

Using Taylor expansion and reserving the linear terms, we get

(6) $$\begin{array}{c} \hat{M}\left(x_{0}\right)\delta \ddot{y}+\hat{C}\left(x_{0}\right)\delta \dot{y}+\hat{K}\left(x_{0},\lambda_{0}\right)\delta y=0, \end{array}$$

where $\hat{M}\left(x_{0}\right)=\left[\begin{array}{cc} M\left(x_{0}\right) & 0\\ 0 & 0 \end{array}\right]$, $\hat{C}\left(x_{0}\right)=\left[\begin{array}{cc} C\left(x_{0}\right) & 0\\ 0 & 0 \end{array}\right]$, $\hat{K}\left(x_{0},\lambda_{0}\right)=\left[\begin{array}{cc} K\left(x_{0},\lambda_{0}\right) & G(x_{0})\\ G^{T}(x_{0}) & 0 \end{array}\right]$, $M\left(x_{0}\right)=\left(\partial F/\partial \ddot{x}\right)\left(x_{0}\right)$, $C\left(x_{0}\right)=\left(\partial F/\partial \dot{x}\right)\left(x_{0}\right)$, **K**(**x**_0_, ***λ***_0_) = (∂(**F** + **G** ***λ***)/∂**x**)(**x**_0_, ***λ***_0_), and $\delta y={\left[\begin{array}{cc} \delta x & \delta \lambda \end{array}\right]}^{T}$. Writing the solution as *δ* **y** = *e* ^*rt*^ **V**, where *r* is one of the eigenvalues, **V** is the corresponding eigenvector and the eigenvalue problem becomes

(7) $$\begin{array}{c} \left(r^{2}\hat{M}+r\hat{C}+\hat{K}\right)V=0. \end{array}$$

This equation represents a standard eigenvalue problem that could be solved using standard solver. The frequency shift could be used to insure that **K** is nonsingular.

Avilés et al. [21] presented a procedure for the solution of the nonlinear static equilibrium problem in complex multibody mechanical systems, including rigid and elastic elements. The error function was simple based on the potential function and set of nonlinear constraints. Lagrange multipliers along with various versions of the augmented Lagrange multipliers were used to form the system dynamic equations. A Newton-Raphson second-order method is used for seeking function minima for equilibrium positions. Wang et al. [22] used the bond graph to improve the efficiency for the kintostatic analysis of complex multibody systems. They proposed an effective decoupling method to simplify the resulting complexity of the equation of motion. As per Giampiero and Manfred [1], the linearized equation of motion will lead to nonlinear equations that can be solved using Newton's method.

Similarly, commercial multibody dynamic simulation solvers like SIMPACK use Newton-Raphson based solver for nonlinear equations and find the system equilibrium positions and/or calculate the preload in the springs and bushing elements. This solution approach has been used for trains, automotive, and airplane systems. Another approach to determine the static equilibrium is the quasistatic approach. In multibody systems, this approach may not be very efficient if there is significant difference in the inertia of the bodies in the system or if there are dynamic oscillators in the system.

This paper describes a general purpose formulation and implementation for modeling rigid and flexible body in multibody system based on the joint coordinates formulation. A new approach for solving static equilibrium will be presented in this paper. The presented static solver utilizes a recursive transient solver to evaluate the system equations of motion. An energy dissipative damping is introduced to eliminate the system oscillations around the equilibrium position. The system states are dampened using weighted damping factor that is function of the state velocity and the state error. The attributes associated with this method are the ability to handle large heterogeneous systems and ability to linearize the system in terms of arbitrary user-defined coordinates in a straightforward implementation. The spatial algebra operators are used to formulate the kinematic and dynamic equations of motion.

This paper is organized as follows. In the following section, the structure of the equation of motion of the multibody system using joint coordinate formulation is introduced for rigid body system based on the spatial algebra operator. In Section 3, the flexible body kinematic and dynamic equations of motion are presented. In Section 4, the equations of motion of the constrained multibody systems with closed loops will be presented.  Section 5 outlines the constraint enforcement technique based on Baumgarte stabilization approach.  Section 6 presents the proposed static equilibrium technique based on the energy drainage.  Section 7 outlines the recursive algorithm implemented to solve the proposed multibody system equations of motion.  Section 8 presents sample results of the proposed static solution approach for multibody systems. Finally, this paper is summarized and some conclusions are drawn in Section 9.

## 2. Multibody Formulation and System Equation of Motion

**Figure 1:**
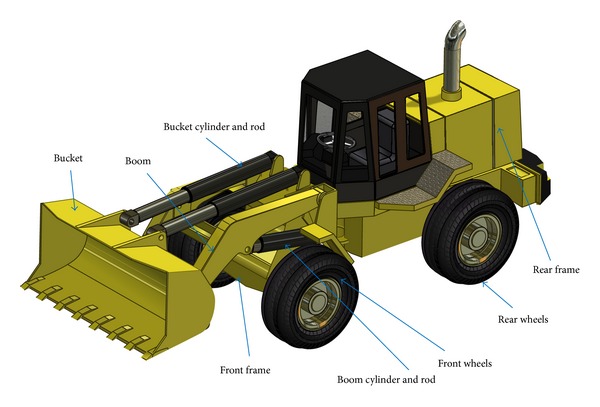
Typical structure of a wheel loader.

Figure 1 shows an illustration of the typical structure of articulated earth-moving equipment, a wheel loader. The major dynamic components of the machine, as shown in the figure, include the rear frame which contains the engine and drive train, the right and left rear wheels which are connected to the rear frame, the front frame which articulates with respect to the rear frame to steer the machine, the boom which is connected to the front frame and is driven by hydraulic cylinders, the bucket which is connected to the boom and is controlled by other hydraulic cylinders, and the left and right front wheels which are connected to the front frames. This machine model will be used to relate the theoretical multibody equations to physical model for clarification.

**Figure 2:**
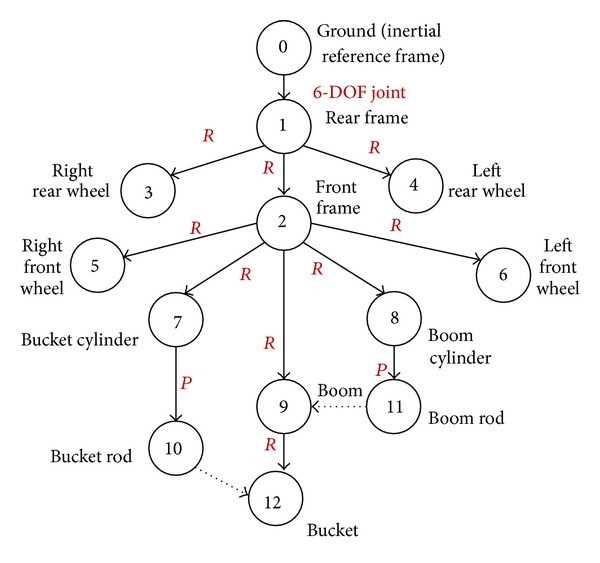
Kinematic tree of the wheel loader model.

The proposed formulation in this investigation utilizes a joint coordinates based multibody approach. The kinematic tree or connectivity graph is developed to describe the system topology based on the connectivity between the different bodies in the system. The ground body (the inertial body) is considered the root (base) of the kinematic tree. Any dynamic body in the system is connected or referenced to one parent body through an arc joint that allows one or more DoF. While each body can have only one parent (ancestor body), it could have one or more child bodies (descendant bodies). In the kinematic tree the root body is numbered as 0 while the descendant bodies are numbered consecutively from 1 to *n*_*b*_. The body and the joint connecting it to its parent are given the same number [10, 23]. It should be mentioned that the kinematic tree is not unique. Using the parent-child relation, a parent-child list could be developed and stored to be used later in the recursive calculations [13, 24, 25]. Any arbitrary body in the system could be modeled as rigid or flexible body.  Figure 2 shows the kinematic tree of the wheel loader under investigation. The dynamic bodies are represented by the circles and the joints are represented by solid arrows. The rear frame is considered the base of our kinematic tree and is referenced to the ground with free joint (joint with 6-DoF). The rear wheels and the front frame are considered descendants of the rear frame and connected to their parent by revolute joints. The remaining bodies in the physical model are shown in the kinematic tree. It should be mentioned that, although the bucket and the boom are driven by two hydraulic cylinders each, only one cylinder is shown in the kinematic tree for simplicity,

(8) $$\begin{array}{c} \text{parent}=\text{list}\left(k\right)=\left\{0,1,1,1,2,2,2,2,2,7,8,9\right\}. \end{array}$$

**Figure 3:**
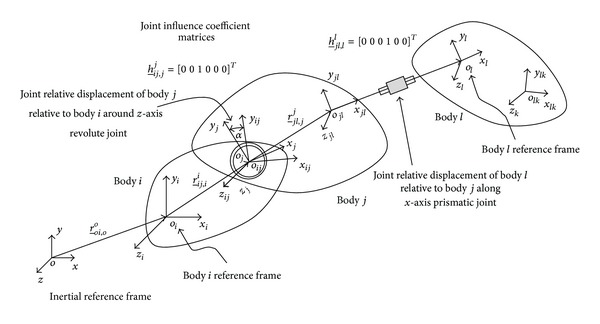
Body kinematics and joint connections.

In the proposed formulation the child body is joined to its parent through two markers; as shown in Figure 3, one marker is attached to the parent side called connector while the other marker is on the child side called the output marker. The output marker is the reference frame of the child body. The body kinematic quantities and dynamic matrices are expressed with respect to the body reference frame. The position and orientation of the connectors are measured relative to the parent reference marker through the spatial transformation matrix, as shown in Figure 3. The joint degrees of freedom, representing the joint variables, are defined as the allowed relative motion between the two connecting markers. Relative motion between the markers could be translation or rotation along one of the connector marker axes. Massless markers could be inserted between the two bodies to represent joints with more than one DoF. The joint variables displacement, velocities, and accelerations are used as the body states. The Cartesian displacements, velocities, acceleration vectors, and the joint reaction forces are considered as augmented algebraic variables.

The position vector of body *i* is defined in the global coordinate system by position vector of the origin of the body reference marker ${\underline{r}}_{0i,i}^{0}$, as shown in Figure 3, while the orientation of the body could be described by the 3 × 3 rotation matrix ${\underline{R}}^{0i}$ which is a function of set of spatial rotational angles. The spatial velocity vector of body *i*, $v_{0i,i}^{0}={\left[\begin{array}{cc} {\underline{\omega }}_{0i}^{0} & {\dot{\underline{r}}}_{0i,i}^{0} \end{array}\right]}^{T}$, could be obtained by differentiating the position vector and the body orientation parameters. As the recursive formulation is based on relative motion, the relative spatial velocity vector of the reference frame of a child body *j* with respect to its parent rigid body *i* observed at the origin of the local coordinate system of body *j* can be written as

(9) $$\begin{array}{c} v_{ij,j}^{j}={\left[\begin{array}{cc} {\underline{\omega }}_{ij}^{j} & {\dot{\underline{r}}}_{ij,j}^{j} \end{array}\right]}^{T}, \end{array}$$

where ${\underline{\omega }}_{ij}^{j}$ is the time derivative of the orientation parameters of body *j* with respect to body *i* and ${\underline{\dot{r}}}_{ij,j}^{j}$ is the derivative of the position vector of body *j* with respect to body *i*. The relative velocity across the joint between body *i* and body *j* can be written as follows:

(10) $$\begin{array}{c} v_{ij,j}^{j}=h_{}^{j}{\dot{q}}_{j}^{}, \end{array}$$

where **h** ^*j*^ is a 6 by *n*_*d*_ joint influence coefficient matrix or partial velocity matrix corresponding to particular columns of the identity matrix depending on which *n*_*d*_ primitive degrees of freedom are represented by **q**_*j*_, as shown in Figure 3, and **q**_*j*_ and ${\dot{q}}_{j}^{}$ are the vector of joint variables and its time derivatives.

The spatial velocity vector can be transformed from the CS at *j* into the CS at *i* using spatial transformation matrix as follows [9, 10, 24]:

(11) $$\begin{array}{c} v^{i}=X_{}^{ij}v^{j}, \end{array}$$

where *X* ^*ij*^ is the spatial transformation matrix and *i* and *j* indicate the coordinate systems. The general transformation matrix *X* ^*ij*^ is the result of spatial rotation followed by a spatial translation as follows:

(12) $$\begin{array}{c} X_{}^{ij}=L_{}^{ij}R^{ij}=\left[\begin{array}{cc} I & 0\\ {\underline{\tilde{r}}}_{ij}^{i} & I \end{array}\right]\left[\begin{array}{cc} {\underline{R}}^{ij} & 0\\ 0 & {\underline{R}}^{ij} \end{array}\right]=\left[\begin{array}{cc} {\underline{R}}^{ij} & 0\\ {\underline{R}}^{ij}{\underline{\tilde{r}}}_{ij}^{i} & {\underline{R}}^{ij} \end{array}\right], \end{array}$$

where *L* ^*ij*^ is the 6 × 6 spatial translation matrix, ${\underline{r}}_{ij}^{i}$ is the 3 × 1 translational displacement vector of CS *j* relative to CS *i* defined in the CS *i*, ${\underline{\tilde{r}}}_{ij}^{i}$ is 3 × 3 skew symmetric matrix representing the cross-product operation of ${\underline{r}}_{ij}^{i}$, *R* ^*ij*^ is the 6 × 6 spatial rotation matrix, and ${\underline{R}}^{ij}$ is the 3 × 3 transformation matrix relating the coordinate frames *i* and *j*.

The spatial velocity vector of body *j* defined in the global coordinate system could be computed as follows:

(13) v0j,00=X0jv0j,jj,where  v0j,jj=Xjiv0i,ii+vij,jj=v0i,jj+hj,jjq˙j.

The spatial velocity vector of a descendant body could be calculated recursively using its parent spatial velocity as follows:

(14) $$\begin{array}{c} v_{0j,0}^{0}=v_{0i,0}^{0}+v_{ij,0}^{0}=v_{0i,0}^{0}+X_{}^{0j}h_{}^{j}{\dot{q}}_{j}. \end{array}$$

Assuming the system shown in Figure 3 is modeled using rigid bodies, the velocity of body *l* could be written in a similar way as follows:

(15) $$\begin{array}{c} v_{0l,l}^{l}=X_{}^{lj}v_{0j,j}^{j}+h_{}^{l}{\dot{q}}_{l}. \end{array}$$

Rearranging the terms in the velocity equations, the recursive form of the system velocity could be expressed as follows [13]:

(16) $$\begin{array}{c} \left[\begin{array}{ccc} I & 0 & 0\\ -X_{}^{ji} & I & 0\\ 0 & -X_{}^{lj} & I \end{array}\right]\left[\begin{array}{c} v_{oi,i}^{i}\\ v_{oj,j}^{j}\\ v_{ol,l}^{l} \end{array}\right]=\left[\begin{array}{ccc} h_{}^{i} & 0 & 0\\ 0 & h_{}^{j} & 0\\ 0 & 0 & h_{}^{l} \end{array}\right]\left[\begin{array}{c} {\dot{q}}_{i}\\ {\dot{q}}_{j}\\ {\dot{q}}_{l} \end{array}\right]. \end{array}$$

The velocity recursive equations in (16) could be written in a compact form as follows:

(17) $$\begin{array}{c} T_{a}^{ll}v_{a}^{l}=H_{a}^{l}{\dot{q}}_{a}=v_{r}^{l}, \end{array}$$

where *T*_*a*_^*ll*^ represents the assembled system topology or connectivity matrix which could be constructed from the connectivity graph, the superscript *l* refers to local matrices and the superscript *a* refers to the system assembly matrix, and **H**_*a*_^*l*^ is the assembly influence coefficient matrices grouped in blocks corresponding to the joints' DoF.

It should be mentioned that *T*_*a*_^*ll*^ is a lower triangular matrix and thus has simple inverse that maintains the topological structure of the original matrix and becomes an upper triangular matrix as follows [26]:

(18) $$\begin{array}{c} {\left(T_{a}^{ll}\right)}^{-1}=\left[\begin{array}{ccc} I & -{\left(X_{}^{ji}\right)}^{T} & 0\\ 0 & I & -{\left(X_{}^{lj}\right)}^{T}\\ 0 & 0 & I \end{array}\right]. \end{array}$$

Similarly the spatial acceleration vector of body *j* could be obtained by differentiating the velocity vector defined in (14) as follows:

(19) a0j,00=a0i,00+aij,00,where, aij,00=hj,00q¨j+v~0j,00hj,00q˙j.

Assuming that the quadratic velocity term could be expressed as $\gamma_{0j,0}^{0}={\tilde{v}}_{0j,0}^{0}h_{j,0}^{0}{\dot{q}}_{j}={\tilde{v}}_{0j,0}^{0}v_{0j,0}^{0}$, the global acceleration vector could written as follows:

(20) $$\begin{array}{c} a_{0j,0}^{0}=a_{0i,0}^{0}+a_{ij,0}^{0}=a_{0i,0}^{0}+h_{j,0}^{0}{\ddot{q}}_{j}+\gamma_{0j,0}^{0}. \end{array}$$

The recursive form to calculate the system local accelerations could be written as follows:

(21) $$\begin{array}{c} T_{a}^{ll}a_{a}^{l}=H_{a}^{l}{\ddot{q}}_{a}+{\dot{H}}_{a}^{l}{\dot{q}}_{a}=H_{a}^{l}{\ddot{q}}_{a}+{\tilde{v}}_{r}^{l}v_{r}^{l}=H_{a}^{l}{\ddot{q}}_{a}+\gamma_{a}^{l}. \end{array}$$

Equations (17) and (21) could be used to recursively calculate the velocity and acceleration of the system bodies marching outward from the root to the descendant bodies. Similarly, the system topological matrix could be used to project the joint forces in the joint subspace as follows:

(22) $$\begin{array}{c} Q_{a}={\left(H_{a}^{l}\right)}^{T}F_{a}^{l}. \end{array}$$

The kinematic equations derived earlier could be used to express the rigid body equations of motion from the momentum equations. The spatial momentum vector of the rigid body *j* can be written as follows:

(23) $$\begin{array}{c} P_{j,G}^{j}=M_{j,GG}^{jj}v_{0j,G}^{j}, \end{array}$$

where **P**_*j*,*G*_^*j*^ is the spatial momentum of body *j*, *M*_*j*,*GG*_^*jj*^ is the spatial mass matrix defined at the body center of mass marker *G*, and **v**_0*j*,*G*_^*j*^ is the global velocity of the marker *G* located at the body center of mass. The centroidal spatial mass matrix is defined as follows:

(24) $$\begin{array}{c} M_{j,GG}^{jj}=\left[\begin{array}{cc} {\underline{J}}_{j,GG}^{jj} & 0\\ 0 & m_{j}\underline{I} \end{array}\right], \end{array}$$

where *m*_*j*_ is the mass body *j*, $\underline{I}$ is 3 × 3 identity matrix, and ${\underline{J}}_{j,GG}^{jj}$ is the 3 × 3 matrix representing the body *j* moment of inertia tensor defined at a marker located at the body center of mass, *G*. This rigid body momentum can be transformed into the global coordinate system and differentiated with respect to time to obtain the body equation of motion as follows:

(25) $$\begin{array}{c} M_{j,00}^{00}a_{0j,0}^{0}-{\tilde{v}}_{0j,0}^{0T}M_{j,00}^{00}v_{0j,0}^{0}=G_{j,0}^{0}, \end{array}$$

where the symbol 0 refers to global coordinate system, $G_{j,0}^{0}={\left[\begin{array}{cc} {\underline{\tau }}_{j,0}^{0} & {\underline{g}}_{j}^{0} \end{array}\right]}^{T}$ is the spatial force vector including the gravitational forces, ${\underline{g}}_{j}^{0}$ is the sum of all external forces acting on the body *j*, and ${\underline{\tau }}_{j,0}^{0}$ contains the sum of all torques and the moments of all forces about the origin of frame 0.

The equation of motion of any two connected bodies *i* and *j* could be driven from the free body diagram as follows. For body *j*, the equation of motion in its local coordinate system could be written as

(26) $$\begin{array}{c} M_{j,jj}^{jj}a_{0j,j}^{j}-F_{j,j}^{j}=G_{j,j}^{j}+{\tilde{v}}_{0j,j}^{jT}P_{j,j}^{j}, \end{array}$$

where **F**_*j*,*j*_^*j*^ is the vector of reaction forces of the joint connecting body *j* to its parent. While for body *i*, the equation of motion in its local coordinate system could be written as

(27) $$\begin{array}{c} M_{i,ii}^{ii}a_{0i,i}^{i}-F_{i,i}^{i}+{\left(X_{}^{ji}\right)}^{T}F_{j,j}^{j}=G_{i,i}^{i}+{\left({\tilde{v}}_{0i,i}^{i}\right)}^{T}P_{i,i}^{i}. \end{array}$$

The equations could be rearranged in matrix form as follows:

(28) [Mi,iiii00Mj,jjjj][a0i,iia0j,jj]+[I−(Xji)T0I][−Fi,ii−Fj,jj]  =[Gi,ii+(v~0i,ii)TPi,iiGj,jj+(v~0j,jj)TPj,jj].

Similar to the velocity and acceleration equations (14), (15), (16), and (17), it could be shown that the connectivity matrix could be used to simplify the expression of the system equations of motion. The system equation of motion could be written for the whole system assembly in a more compact form as follows:

(29) $$\begin{array}{c} M_{}^{ll}a_{a}^{l}-{\left(T_{a}^{ll}\right)}^{T}F_{a}^{l}=G_{a}^{l}+{\left({\tilde{v}}_{a}^{l}\right)}^{T}P. \end{array}$$

The system of equations in (29) contains the unknown Cartesian acceleration of the bodies as well as the joint reaction forces. In order to be able to solve the equation of motion, the kinematic relations from (21), the constraint equations from (22), and the system dynamic equation (29) are rearranged in matrix form as follows:

(30) $$\begin{array}{c} \left[\begin{array}{ccc} M_{}^{ll} & 0 & {\left(T_{a}^{ll}\right)}^{T}\\ 0 & 0 & -H_{a}^{l}\\ T_{a}^{ll} & -{\left(H_{a}^{l}\right)}^{T} & 0 \end{array}\right]\left[\begin{array}{c} a_{a}^{l}\\ {\ddot{q}}_{a}\\ -F_{a}^{l} \end{array}\right]=\left[\begin{array}{c} G_{a}^{l}+{\left({\tilde{v}}_{a}^{l}\right)}^{T}P_{a}^{l}\\ Q_{a}\\ \gamma_{a}^{l} \end{array}\right], \end{array}$$

where *M* ^*ll*^ is a block diagonal inertia matrix expressed in the Cartesian form, *T*_*a*_^*ll*^ is the body connectivity matrix, *H*_*a*_^*l*^ is a block diagonal joint influence coefficient matrix, *a*_*a*_^*l*^ is the body Cartesian accelerations, **F**_*a*_^*l*^ is a vector of the Lagrange multipliers, ${\ddot{q}}_{a}^{l}$ is the second time derivative of joint variables, **G**_*a*_^*l*^ is a vector of the externally applied loads, **γ**_*a*_^*ll*^ is the quadratic constraint derivatives, and *Q*_*a*_^*l*^ is the generalized forces. Since the inertia matrix *M* ^*ll*^ is invertible, (30) could be solved to get the unknown joint accelerations ${\ddot{q}}_{a}$ which can be used to calculate the joint Cartesian accelerations, **a**_*a*_^*l*^, and the joint reaction forces **F**_*a*_^*l*^ as follows:

(31) ((Hal)T(Tall)−TMll(Tall)−1Hal)q¨a =Qa+(Hal)T(Tall)−T(Gal+(v~al)TPal).

Equation (31) could be written in a compact form as follows:

(32) $$\begin{array}{c} M_{qq}^{}{\ddot{q}}_{a}=Q, \end{array}$$

where **M**_*qq*_ is the generalized mass matrix projected in the joint subspace and can be calculated as follows:

(33) $$\begin{array}{c} M_{qq}^{}={\left(H_{a}^{l}\right)}^{T}{\left(T_{a}^{ll}\right)}^{-T}M_{}^{ll}{\left(T_{a}^{ll}\right)}^{-1}H_{a}^{l} \end{array}$$

and **Q** is the generalized force vector projected in the joint subspace and can be calculated as follows:

(34) $$\begin{array}{c} Q=Q_{a}+{\left(H_{a}^{l}\right)}^{T}{\left(T_{a}^{ll}\right)}^{-T}\left(G_{a}^{l}+{\left({\tilde{v}}_{a}^{l}\right)}^{T}P_{a}^{l}\right). \end{array}$$

This equation of motion (32) represents minimum set of differential equations that are function of the system generalized coordinates. However, this form is limited to open-loop unconstrained mechanisms and therefore is suitable only for robotic type mechanisms.

## 3. Equation of Motion of Rigid and Flexible Body System

The flexible body dynamics can be modeled in the multibody system as a reduced form of the finite element model. The finite element can be reduced using nodal approach or modal approach. Modal formulation uses a set of kinematically admissible modes to represent the deformation of the flexible body [27, 28]. Component mode synthesis approach is widely used in the multibody dynamics codes to accurately simulate the flexible body dynamics in the multibody system. The main advantage of the modal formulation is that fewer elastic modes can be used to accurately capture the flexible body dynamics at reasonable computational cost [29, 30]. Craig-Bampton approach was introduced to account for the effect of boundary conditions and the attachment joints of the flexible body [31, 32]. The structure of the reduced flexible body equation-of-motion can be written as follows [23]:

(35) $$\begin{array}{c} \left[\begin{array}{cc} M_{rb}^{rr} & M_{}^{rf}\\ M_{}^{fr} & M_{}^{ff} \end{array}\right]\left[\begin{array}{c} a_{}^{r}\\ {\ddot{q}}_{}^{f} \end{array}\right]=\left[\begin{array}{c} G_{}^{r}\\ Q_{}^{f} \end{array}\right], \end{array}$$

where *r* refers to the flexible body's local reference frame, *f* refers to flexible or elastic coordinates, *M*_*rb*_^*rr*^ represents the 6 × 6 inertia matrix associated with the reference motion, *M* ^*rf*^, *M* ^*fr*^ are the inertia coupling terms between the reference motion and the elastic coordinates, *M* ^*ff*^ is the inertia matrix associated with the elastic coordinates, *a* ^*r*^ is the reference frame acceleration in the Cartesian space, *q* ^*f*^ represents the elastic displacements measured relative to that frame, *G* ^*r*^ is the vector of external forces including centrifugal and Coriolis forces [1, 23, 27], and *Q* ^*f*^ is the vector of elastic forces projected into the modal space. It should be mentioned that the matrices *M*_*rb*_^*rr*^, *M* ^*rf*^, and *M* ^*fr*^ depend on the elastic coordinates and should be updated every time step. To improve the computation efficiency of the flexible body simulation, a set of inertia shape invariants (ISI) are identified and calculated before the simulation starts. The ISI are used during the dynamic simulation to update the inertia terms. The ISI depend on the kinematic expression of the system equations.

A general form of the equation of motion of multibody system with flexible bodies could be written in a compact form as follows [23]:

(36) $$\begin{array}{c} \left[\begin{array}{ccc} M_{}^{rr} & T_{}^{T} & M_{}^{rf}\\ T_{}^{} & 0 & -H_{}^{}\\ {\left(M_{}^{rf}\right)}^{T} & -H_{}^{T} & M_{}^{ff} \end{array}\right]\left[\begin{array}{c} a\\ -F\\ \ddot{q} \end{array}\right]=\left[\begin{array}{c} G\\ \gamma \\ Q \end{array}\right], \end{array}$$

where the block diagonal matrix **M** ^*rr*^ is composed of six by six inertia matrices that represent the Cartesian inertia matrices associated with the reference frame for the rigid and flexible bodies in the system, **M** ^*rf*^ is the inertia coupling terms between the modal elastic coordinates and Cartesian reference accelerations, **M** ^*ff*^ is a block diagonal matrix containing the flexible body inertias, **T** is a lower triangular topology matrix, **H** is a block matrix of the joint influence coefficient matrices, **a** is the vector of Cartesian accelerations, **F** is the vector of joint reaction forces, $\ddot{q}$ is the vector of joint accelerations with the appended elastic coordinates, **G** is the vector of external forces, **γ** is a column matrix of 6 × 1 spatial quadratic acceleration vectors, and **Q** is the vector of joint and elastic forces.

The system of equations given by (36) can be easily solved for the joint accelerations and the modal coordinates accelerations. Then the Cartesian accelerations and the reaction forces could be easily computed. Since the inverse of system topology matrix **T** is just its transpose, manipulating the terms in (36), we can get the joint and modal accelerations as follows:

(37) (Mff+HT(TTMrrT)H+HTTTMrf+MfrTH)q¨  =Q+HTTTG−HT(TTMrrT)γ−MfrTγ.

This equation could be written in a compact form as follows:

(38) $$\begin{array}{c} M_{qq}{\ddot{q}}_{a}=Q. \end{array}$$

The Cartesian accelerations could be calculated as follows:

(39) $$\begin{array}{c} a=TH\ddot{q}+T\gamma , \end{array}$$

and the Cartesian joint reaction forces could be obtained from

(40) $$\begin{array}{c} F=T^{T}M^{rf}+\left(T^{T}M^{aa}T\right)H\ddot{q}-T^{T}G+\left(T^{T}M^{aa}T\right)\gamma . \end{array}$$

The sequence of evaluating the terms in (37) to (40) could be optimized in order to minimize the computational efforts and to avoid repeated calculations. Both (32) and (38) represent the minimum set of differential equations that can completely describe the system dynamics.

## 4. Equation of Motion of Constrained System

The abovementioned development could be efficiently used to model open-loop systems like robots and human body systems. In the open-loop systems, each body is connected with one joint only. This open-loop systems approach will not be adequate to model the wheel loader system shown in Figure 1. In the kinematic tree, shown in Figure 2, the solid arrows represent a joint where the dotted lines represent a joint that will form a closed kinematic loop. According to this description, the kinematic tree of the wheel loader contains two closed loops formed of the following list of bodies: Γ^1^ = {2,8, 11,9} and Γ^2^ = {2,9, 12,10,7}. Each of those two loops could be represented by a set of constraint equations that are function of the joint variable of all the bodies forming the loop as follows: Φ^1^(**q**_2_, **q**_8_, **q**_11_, **q**_9_) = 0 and Φ^2^(**q**_2_, **q**_9_, **q**_12_, **q**_10_, **q**_7_) = 0. The first and second derivatives of the constraint equations could be easily evaluated from the kinematic equations. The multibody system's equations of motion with appended constraints could be formed as follows:

(41) $$\begin{array}{c} \left[\begin{array}{cc} M_{qq}^{} & J^{T}\\ J & 0 \end{array}\right]\left[\begin{array}{c} {\ddot{q}}_{a}\\ \lambda_{q} \end{array}\right]=\left[\begin{array}{c} Q\\ \gamma_{q} \end{array}\right], \end{array}$$

where **M**_*qq*_ is the generalized mass matrix from (32) and (38), **J** is the Jacobian matrix, and the other column matrices are similar to those described earlier.

The coefficient matrix in the left hand side of (41) is often singular, and general-purpose sparse matrix solver algorithms are routinely used to analyze, factor, and solve the equations. An alternative approach based on generalized coordinate partitioning [14] may be employed at this stage. The factored forms of (33) and (34) used to obtain (32) play a significant role in the solver efficiency. The generalized coordinate partitioning method [14] first analyzes the model topology and decomposes it into a number of kinematically uncoupled superelements, each with a small Jacobian matrix [2]. The uncoupled acceleration constraint equations could be arranged as follows:

(42) $$\begin{array}{c} \left[\begin{array}{ccc} \Phi_{q}^{1} & \cdots & 0\\ \vdots & \vdots & \vdots \\ 0 & \cdots & \Phi_{q}^{n} \end{array}\right]\left[\begin{array}{c} {\ddot{q}}^{1}\\ \vdots \\ {\ddot{q}}^{n} \end{array}\right]=\left[\begin{array}{c} \gamma^{1}\\ \vdots \\ \gamma^{n} \end{array}\right]. \end{array}$$

The Jacobian matrices in (42) are factored into LU form using complete pivoting to partition the variables into dependent and independent sets. The results are then combined into a revised set of constraint equations [33]

(43) $$\begin{array}{c} {\ddot{q}}^{d}=B{\ddot{q}}^{i}+\gamma^{d}, \end{array}$$

where ${\ddot{q}}^{d}$ is the vector of dependent variables and ${\ddot{q}}^{i}$ is the vector of independent variables. Appending (43) with a set of Lagrange multipliers to the partitioned and permuted equations of motion gives

(44) $$\begin{array}{c} \left[\begin{array}{ccc} M_{q}^{dd} & I & M_{q}^{di}\\ I & 0 & -B\\ M_{q}^{id} & -B^{T} & M_{q}^{ii} \end{array}\right]\left[\begin{array}{c} {\ddot{q}}^{d}\\ \lambda^{d}\\ {\ddot{q}}^{i} \end{array}\right]=\left[\begin{array}{c} Q^{d}\\ \gamma^{d}\\ Q^{i} \end{array}\right]. \end{array}$$

The coefficient matrix in the upper-left part of (44) is structurally nonsingular, so it can be reduced to

(45) $$\begin{array}{c} {\overset{-}{M}}_{q}^{ii}{\ddot{q}}^{i}={\overset{-}{Q}}^{i}, \end{array}$$

where

(46) M−qii=Mqii+MqidB+BTMqdi+BTMqddB,Q−i=(Qi−Mqidγd)+BT(Qd−Mqddγd).

Equation (45) could be solved by Cholesky factorization.

## 5. Constraint Enforcement

The equations of motion in (41) and (44) are index-2 differential algebraic equations (DAE) [1]. This set of DAE are traditionally linearized and solved by implicit numerical integrators [34–36]. The generalized coordinate partitioning approach [14] is used to avoid the complexity and cost of implicit solvers, but this approach does not guarantee continued satisfaction of the system constraints. Baumgarte [37] introduced a constraint stabilization method based on the control theory. In this approach, the original acceleration level constraints are replaced with

(47) $$\begin{array}{c} \ddot{\Phi }+\alpha \dot{\Phi }+\beta \Phi =0, \end{array}$$

where *α* and *β* are often chosen to appropriately achieve critical damping. The Baumgarte stabilization is implemented in the following form:

(48) $$\begin{array}{c} \ddot{\Phi }+2\omega_{n}\dot{\Phi }+\omega_{n}^{2}\Phi =0, \end{array}$$

where *ω*_*n*_ is a user-specified gain (natural frequency). Unfortunately, the value of the parameter *ω*_*n*_ is problem dependent, and no general procedure exists for its determination. If $\Phi =\dot{\Phi }=\ddot{\Phi }=0$ represents the constraint equations associated with (41), then substituting (48) into (41) leads to

(49) $$\begin{array}{c} \left[\begin{array}{cc} M_{q} & J^{T}\\ J & 0 \end{array}\right]\left[\begin{array}{c} \ddot{q}\\ \lambda_{q} \end{array}\right]=\left[\begin{array}{c} Q\\ \gamma_{q}-2\omega_{n}\dot{\Phi }-\omega_{n}^{2}\Phi \end{array}\right]. \end{array}$$

A similar modification would be made to the right hand side of (44). The value set for *ω*_*n*_ is critical and affects the integrator performance. If *ω*_*n*_ is too large, constraint violations will be very small, but the equation of motion will tend to be stiff and the integrator will be slow. If *ω*_*n*_ is too small, the stabilization algorithm will not efficiently reduce the constraint violations. From experience, the maximum value of best *ω*_*n*_ is probably near the highest system frequencies in the system. To this end, the developed set of dynamic equations of motion is sufficient to perform the dynamic simulation of open- and closed-loop systems. It is also sufficient for modeling rigid and flexible bodies in the multibody system.

## 6. Static Equilibrium Analysis

The mechanical system can be considered at static equilibrium if the kinetic energy is zero or constant target value and the potential energy is minimum. Many methods have been proposed to solve static equilibrium in nonlinear multibody system models. Most of those proposed techniques require minimizing potential functions and/or linearizing the equations of motion [36] as mentioned earlier. Writing potential functions for arbitrary nonlinear force modules or linearizing them is a daunting task. The problem becomes more challenging when models contain friction and other history-based force models because unique iterative static solutions may not exist. The proposed approach uses the dynamic model itself to solve the static equilibrium position. This approach may have some drawbacks because convergence can be slow in lightly damped systems. In order to improve the solver efficiency, an energy-drainage mechanism is used.

In the energy-drainage approach, a technique similar to the Baumgarte equation was implemented into the solver. After solving (45) to obtain the accelerations $\ddot{q}$, the solver imposes the additional requirement on the second derivatives

(50) $$\begin{array}{c} {\ddot{q}}_{d}=-2\omega_{d}{\dot{q}}_{i}-\omega_{d}^{2}\left(q_{i}-q_{i-1}\right), \end{array}$$

where *ω*_*d*_ is a natural damping frequency and *q*_*i*_ and *q*_*i*−1_ represent the system states at times *t*_*i*_ and *t*_*i*−1_. Choosing *ω*_*d*_ is tricky as it was in the constraint stabilization. If *ω*_*d*_ is too small, the damping will be weak and the model will oscillate too long. And if *ω*_*d*_ is too large, damping will be strong and the model will drift slowly toward equilibrium. With this damping method, equilibrium is approached as ||*q*_*i*_ − *q*_*i*−1_|| gets acceptably small, not necessarily when the kinetic energy gets small.

Another feature of energy-drainage damping is its ability to inhibit the effects of bad initial conditions that often cause models to fly apart and the numerical integrator to fail. Extremely high forces from the offending contact models can cause unrealistic accelerations, which are then integrated to give unrealistic velocities and displacements. When energy-drainage damping is active, the resulting large system velocities are immediately fed back through the above damping equation, and this generates large opposing acceleration terms, canceling those from the model. With energy-drainage damping, the contacting bodies will tend to drift slowly apart until the forces and accelerations become reasonable.

## 7. Recursive Algorithm

At the beginning of the dynamic simulation, the structure of the equation of motion is determined based on a preliminary analysis of the system topology. The dependent and independent variable sets are determined using the generalized coordinate portioning approach. The solver integrates both dependent and independent variable sets and the kinematic constraints are enforced using Baumgarte stabilization, as explained in the previous section. The input states to the integrator represent the first and second time derivative of the joint variables and the output states are the joint displacements and the joint velocities. The recursive algorithm of the multibody dynamic simulation can be summarized as follows.

1. Using the joint variables which returned from the integrator, the solver calculates the Cartesian displacements, velocities, and accelerations. Forward evaluation scheme is utilized (starting from the root body to the descendant/branch bodies).
2. Update and factor the Jacobian matrix.
3. Apply generalized coordinate portioning technique to determine the quality of the independent and dependent set of variables.
4. Calculate the constraint violations and apply the Baumgarte stabilization.
5. Calculate the internal and external forces and apply them to the different bodies (interaction forces between bodies, driver forces, soil/terrain forces, etc.).
6. Transform the inertia matrices into global coordinate system.
7. Calculate the inertia forces and centrifugal and Coriolis forces in Cartesian space.
8. Propagate the external and inertia forces from the descendant bodies into their parents.
9. Project the Cartesian forces into the body joint space.
10. Factor the mass matrix based on the current independent and dependent variables selection.
11. Calculate second derivatives of the joint variables.
12. Apply the energy drainage operator according to   (50).
13. Send the states to the integrator.

The solver utilizes a predictor-corrector integrator with variable-order interpolation polynomials and variable time step. This explicit integrator insures the stability of the solution and the ability to capture the high-speed impacts between the different machine parts.

## 8. Examples

**Figure 4:**
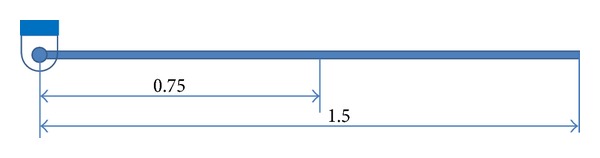
Single pendulum beam.

This section presents some examples of the simulation results multibody system ranging from simple pendulum to a full wheeled machine. The first example represents a uniform beam connected to the ground with a revolute joint to form a single pendulum, as shown in Figure 4. The link has mass of 1.7595 Kg. The beam center of mass is located in the middle. The pendulum was originally horizontal and is allowed to fall under the effect of gravity. The static equilibrium position of this pendulum is known to be vertical.

The proposed multibody formulation and solver were used to model the pendulum and compute the static equilibrium position. The transient simulation is run for a few iterations to allow the system to pick up some kinetic energy. Once the pendulum starts to move the energy draining mechanism is applied. A stopping criterion is set to be a target value of the kinetic energy. When the pendulum reaches this target value, it would have reached the static equilibrium.

**Figure 5:**
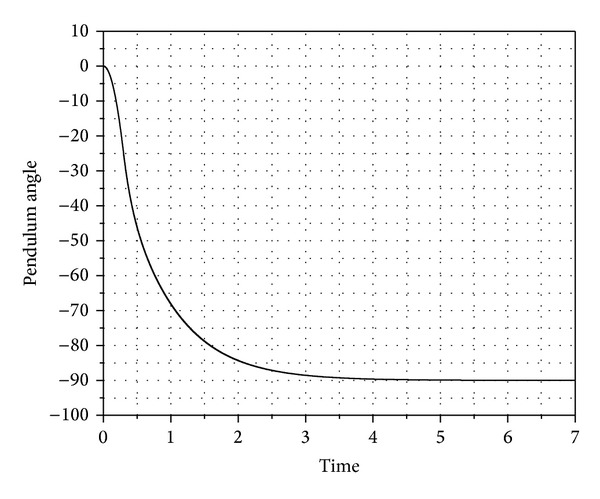
The pendulum angle as it falls under the effect of gravity.

**Figure 6:**
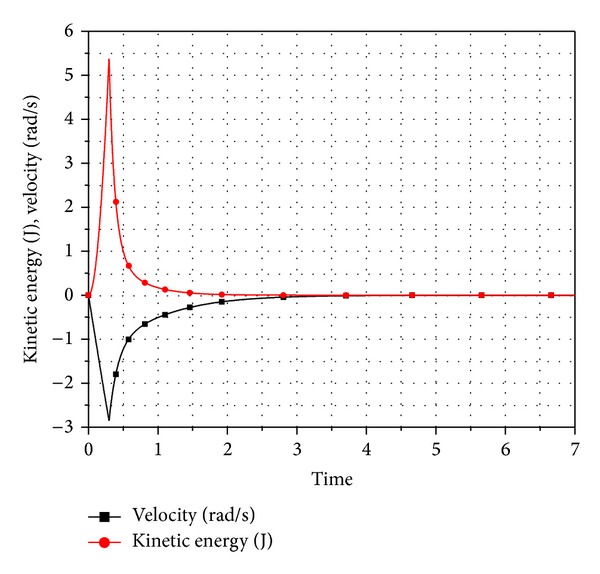
The velocity and kinetic energy of the pendulum.

Figure 5 shows the value of the pendulum angle as it falls under the effect of gravity till the system reaches equilibrium position. The kinetic energy is drained out from the system as shown in Figure 6.

**Figure 7:**
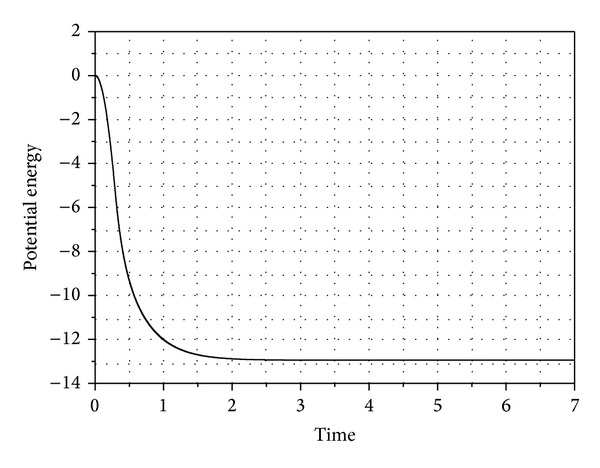
The potential energy of the pendulum.

The change in the pendulum potential energy from the reference is shown in Figure 7. As the pendulum starts moving, the potential energy of the pendulum decreases and the minimum value is reached at the static equilibrium position. The final value of the potential energy is −12.9455J which matches the closed form solution for the pendulum.

It should be mentioned that time in the plots is only a representation of the number of iteration to achieve solution convergence. Also, it should be mentioned that this example represents an extreme case where the pendulum initial position was extremely far from the equilibrium position. This example shows that the solver is stable and can achieve realistic solutions even in extreme cases.

**Figure 8:**
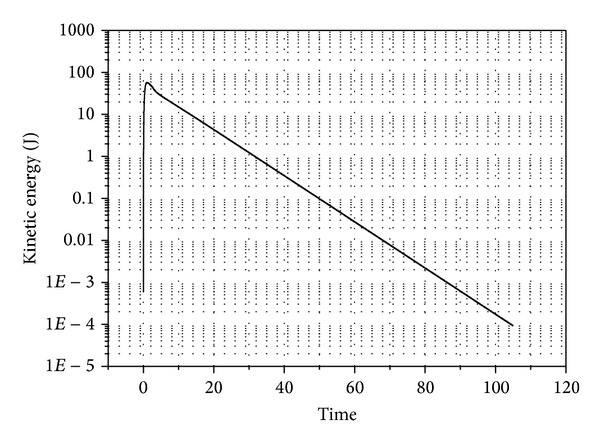
Kinetic energy of the 50-link chain.

**Figure 9:**
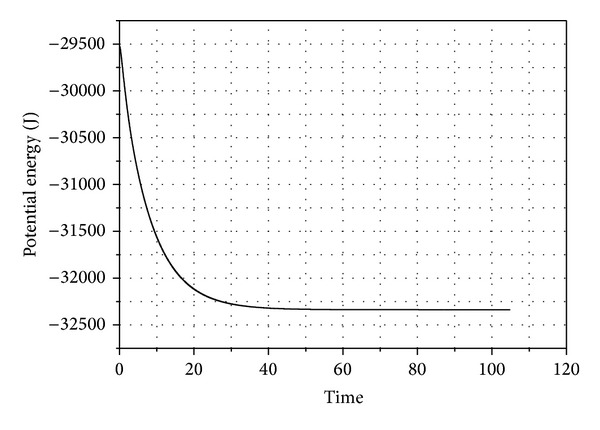
Potential energy of the 50-link chain.

**Figure 10:**
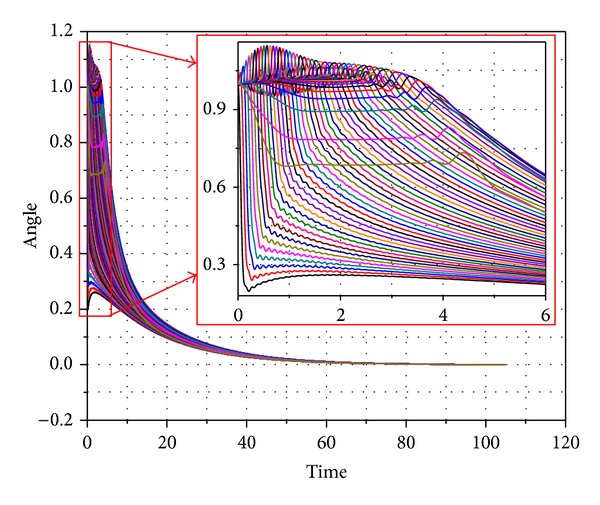
Angles of the links in the 50-link chain.

The second example represents a system that might have minor assembly errors or constraint violations. The system represents a chain of 50 links connected with revolute joints. The chain links had same properties of the link in first example. In this example, the first link is attached to the ground and it has initial angle of 5° from the vertical position. Every following link has an initial deviation of 1° relative to its parent. The static equilibrium analysis was performed on the system. The kinetic energy of the system is shown in Figure 8. The potential energy of the system is shown in Figure 9 while the angles of all the links are shown in Figure 10.

**Figure 11:**
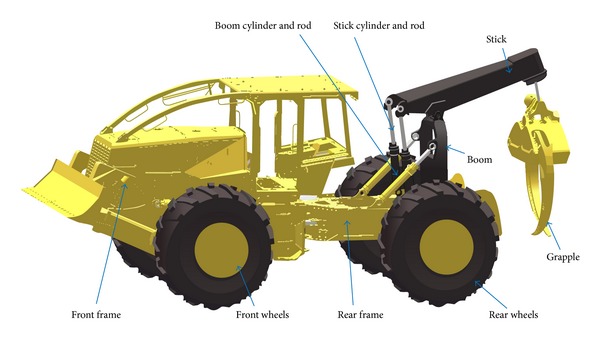
Grapple skidder forestry machine.

To demonstrate the completeness of the proposed approach a full machine with closed kinematic loops will be presented in the following example. Forestry equipment is typically required to work on different terrains ranging from swampy, compactable, and hard soil. It may be required to work on slopes dragging wood logs and tree. During operation and articulations, the location of the center of mass of such machine may change and causes the machine to slip, flip over, or fall. Design of such machines requires accurate prediction of the machine stability under the different loading conditions and at different articulation angles. Also, power requirements analysis of such machine requires the simulation to start from a stable static equilibrium position. A grapple skidder machine, shown in Figure 11, will be used as a demonstration example.

**Figure 12:**
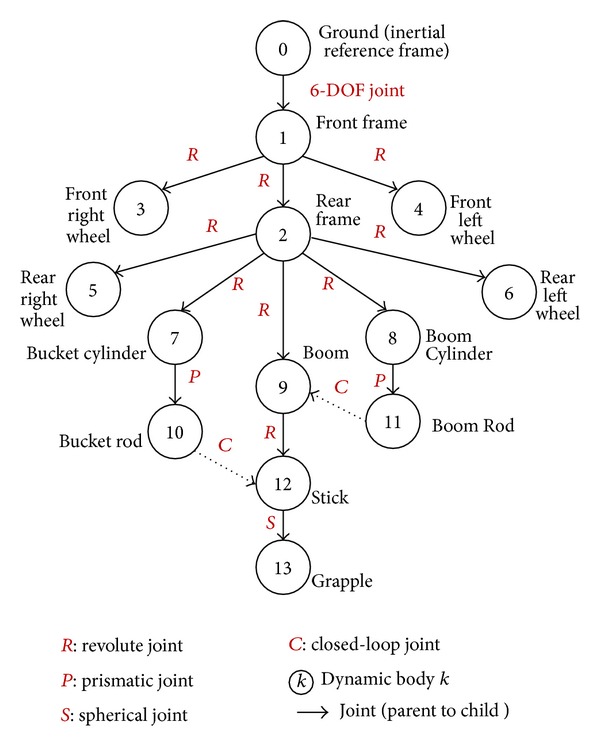
Kinematic tree of log dragging forestry equipment.

The kinematic tree of the machine could be developed, as shown in Figure 12, in order to establish the parent child list. It could be seen that the kinematic tree of grapple skidder is very similar to that of the wheel loader shown in Figure 2. The solver algorithm analyzes the kinematic tree to identify the closed loops of the mechanism and define the constraint equations associated with the closed-loop joints. The constraint equations of the two kinematic closed loops could be written as Φ^1^(**q**_2_, **q**_8_, **q**_11_, **q**_9_) = 0 and Φ^2^(**q**_2_, **q**_9_, **q**_12_, **q**_10_, **q**_7_) = 0.

The next step for the solver is to analyze the closed loops and merge any two loops that share any joint variable into one larger superelement. This operation could be iterative and may lead to very large superelements. In our example, since the two closed loops are sharing the variables of body 9, they have to be merged into a large superelement that contains all the bodies of the two loops. The Jacobian matrix of this superelement could be calculated as follows:

(51) $$\begin{array}{c} J=\left[\begin{array}{ccccccc} \frac{\partial \Phi^{1}}{\partial q_{2}} & \frac{\partial \Phi^{1}}{\partial q_{8}} & \frac{\partial \Phi^{1}}{\partial q_{11}} & \frac{\partial \Phi^{1}}{\partial q_{9}} & 0 & 0 & 0\\ \frac{\partial \Phi^{2}}{\partial q_{2}} & 0 & 0 & \frac{\partial \Phi^{2}}{\partial q_{9}} & \frac{\partial \Phi^{2}}{\partial q_{12}} & \frac{\partial \Phi^{2}}{\partial q_{10}} & \frac{\partial \Phi^{2}}{\partial q_{7}} \end{array}\right]. \end{array}$$

The first raw block of the Jacobian matrix represents the partial derivatives of the first kinematic loop constraint equations with respect to the joint variables of the bodies in the first loop while the second row block represents the partial derivatives of the second loop constraint equations. The system topology matrix will be generated by the solver and should be as follows:

(52) Tall=[I−X2,1I−X3,10I−X4,100I−X2,1−X5,200I−X2,1−X6,2000I−X2,1−X7,20000I−X2,1−X8,200000I−X2,1−X9,2000000I−X2,1−X7,20000−X10,700I−X2,1−X8,200000−X11,800I−X2,1−X9,2000000−X12,900I−X2,1−X9,2000000−X12,900−X13,12I].

The system assembly influence coefficient matrix could be written as follows:

(53) $$\begin{array}{c} H_{a}^{l}=\left[\begin{array}{ccccccccccccc} h^{1} & 0 & 0 & 0 & 0 & 0 & 0 & 0 & 0 & 0 & 0 & 0 & 0\\ 0 & h^{2} & 0 & 0 & 0 & 0 & 0 & 0 & 0 & 0 & 0 & 0 & 0\\ 0 & 0 & h^{3} & 0 & 0 & 0 & 0 & 0 & 0 & 0 & 0 & 0 & 0\\ 0 & 0 & 0 & h^{4} & 0 & 0 & 0 & 0 & 0 & 0 & 0 & 0 & 0\\ 0 & 0 & 0 & 0 & h^{5} & 0 & 0 & 0 & 0 & 0 & 0 & 0 & 0\\ 0 & 0 & 0 & 0 & 0 & h^{6} & 0 & 0 & 0 & 0 & 0 & 0 & 0\\ 0 & 0 & 0 & 0 & 0 & 0 & h^{7} & 0 & 0 & 0 & 0 & 0 & 0\\ 0 & 0 & 0 & 0 & 0 & 0 & 0 & h^{8} & 0 & 0 & 0 & 0 & 0\\ 0 & 0 & 0 & 0 & 0 & 0 & 0 & 0 & h^{9} & 0 & 0 & 0 & 0\\ 0 & 0 & 0 & 0 & 0 & 0 & 0 & 0 & 0 & h^{10} & 0 & 0 & 0\\ 0 & 0 & 0 & 0 & 0 & 0 & 0 & 0 & 0 & 0 & h^{11} & 0 & 0\\ 0 & 0 & 0 & 0 & 0 & 0 & 0 & 0 & 0 & 0 & 0 & h^{12} & 0\\ 0 & 0 & 0 & 0 & 0 & 0 & 0 & 0 & 0 & 0 & 0 & 0 & h^{13} \end{array}\right]. \end{array}$$

When the solver starts, the Cartesian velocities and accelerations are calculated using forward substitution from the root body to the descendant bodies. The Jacobian matrices are updated based on the new values of the spatial displacements and velocities. The external forces acting on the system, the contact forces between the different bodies, and the interaction forces between the system and terrain are calculated and applied to each body's force vector. The system topology matrix is used to project the Cartesian quantities into the joint subspace and define the minimum set of the system variables as shown in (32) to (34). The system Jacobian matrix is then appended to the inertia matrix to form the augmented constrained multibody equation of motion. The solver then utilizes the generalized coordinate portioning approach to identify the dependent and independent variables by factoring the Jacobian matrix into LU form. The independent variable is identified, as shown in (45). The constraint violation penalty is applied to the system equation of motion using Baumgarte equation, as shown in (48). The entries to the mass matrix are permuted based on the order in the LU factorization order.

To perform the static analysis, at this point, the system accelerations are modified using the energy drainage function as shown in (50), and both the dependent and independent variables are sent to the integrator. The integrated states are used to calculate the Cartesian position, velocities, and accelerations of all bodies. The Cartesian velocities are used to calculate the kinetic energy of the system and the global position of the bodies is used to calculate the potential energies. If the value of the kinetic energy of the system reached the target, the solver stops and reports the current state as the body equilibrium position.

**Figure 13:**
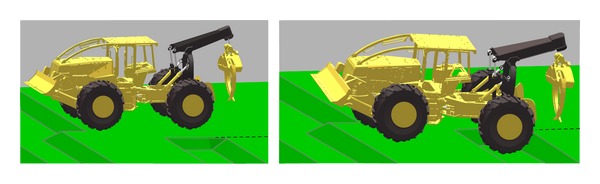
Static equilibrium of wheeled machine (grapple skidder) on a washboard surface.

Figure 13 shows a successful simulation to achieve the static equilibrium of wheeled machine. The machine is dropping from a small height over a washboard surface. As the machine drops, the rear tires fall on the inclined plane of the surface causing the machine to roll down and backward. As the machine contacts the opposite surface of the washboard ditch, the machine comes to static equilibrium.

## 9. Conclusions

This paper presented an approach for evaluating the static equilibrium position of multibody systems. The multibody system can include rigid and flexible bodies. The proposed approach is implemented in a joint-based multibody dynamics formulation that uses the spatial algebra to derive the equation of motion of the multibody system. The kinematic equation of the closed-loop systems was optimized to employ Baumgarte constraint stabilization approach to eliminate the constraint violation while at the same time avoiding using iterative constraint enforcement schemes. In order to determine the system static equilibrium position, an energy drainage mechanism was introduced to modify the system states before integrating it. The static equilibrium is achieved by running a transient simulation of the dynamics system while the integrated states were dampened with the energy drainage operator.

## References

[1] Giampiero M, Manfred P (2004). *Simulation Algorithms in Vehicle System Dynamics*.

[2] Omar MA (2009). An applied approach for large-scale multibody dynamics simulation and machine-terrain interaction. *SAE International Journal of Passenger Cars—Electronic and Electrical Systems*.

[3] Wong JY (1997). Dynamics of tracked vehicles. *Vehicle System Dynamics*.

[4] Lee HC, Choi JH, Shabana AA (1998). Spatial dynamics of multibody tracked vehicles part II: contact forces and simulation results. *Vehicle System Dynamics*.

[5] Ma Z-D, Perkins NC (2002). A track-wheel-terrain interaction model for dynamic simulation of tracked vehicles. *Vehicle System Dynamics*.

[6] Ma Z-D, Perkins NC (2003). An efficient multibody dynamics model for internal combustion engine systems. *Multibody System Dynamics*.

[7] Roland P, Daniel D, Emilio S, Miguel ÁN, Samin JC, Fisette P Validation of a multibody model for an X-by-wire vehicle prototype through field testing.

[8] Shabana AA (2005). *Dynamics of Multibody Systems*.

[9] Nikravesh PE (2005). *An Overview of Several Formulations for Multibody Dynamics*.

[10] Featherstone R (2008). *Rigid Body Dynamics Algorithms*.

[11] Featherstone R (2001). The acceleration vector of a rigid body. *International Journal of Robotics Research*.

[12] Featherstone R, Orin D Robot dynamics: equations and algorithms.

[13] Featherstone R (2005). Efficient factorization of the joint-space inertia matrix for branched kinematic trees. *International Journal of Robotics Research*.

[14] Wehage RA, Haug EJ (1982). Generalized coordinate partitioning for dimension reduction in analysis of constrained dynamic systems. *Journal of Mechanical Design*.

[15] Wehage RA, Belczynski MJ High resolution vehicle simulations using precomputer coefficients.

[16] Rodriguez G, Jain A, Kreutz-Delgado K (1992). Spatial operator algebra for multibody system dynamics. *Journal of the Astronautical Sciences*.

[17] Rodriguez G, Jain A, Kreutz-Delgado K (1991). Spatial operator algebra for manipulator modeling and control. *International Journal of Robotics Research*.

[18] Eich-Soellner E, Führer C (1998). *Numerical Methods in Multibody Dynamics*.

[19] Yang C, Cao D, Zhao Z, Zhang Z, Ren G (2012). A direct eigenanalysis of multibody system in equilibrium. *Journal of Applied Mathematics*.

[20] García de Jalón J, Bayo E (1994). *Kinematic and Dynamic Simulation of Multibody Systems, the Real-Time Challenge*.

[21] Avilés R, Ajuria G, Gómez-Garay V, Navalpotro S (2000). Comparison among nonlinear optimization methods for the static equilibrium analysis of multibody systems with rigid and elastic elements. *Mechanism and Machine Theory*.

[22] Wang ZS, Tao YY, Wen QY (2013). A vector bond graph method of kineto-static analysis for spatial multibody systems. *Applied Mechanics and Materials*.

[23] Omar MA (2014). Modeling flexible bodies in multibody systems in joint-coordinates formulation using spatial algebra. *Advances in Mechanical Engineering*.

[24] Wehage RW Automated procedures for robust and efficient solution of over-constrained multibody dynamics.

[25] Nikravesh PE (1994). Construction of the equations of motion for multibody dynamics using point and joint coordinates. *Computer-Aided Analysis of Rigid and Flexible Mechanical Systems*.

[26] Jain A (2012). Multibody graph transformations and analysis—part I: tree topology systems. *Nonlinear Dynamics*.

[27] Omar MA (2010). *Finite Element Modeling of Leaf Springs in Multibody Systems*.

[28] Omar MA, Shabana AA, Mikkola A, Loh W-YI, Basch R (2004). Multibody system modeling of leaf springs. *Journal of Vibration and Control*.

[29] Bathe KJ (1996). *Finite Element Procedures*.

[30] Hughes TJR (2000). *The Finite Element Method: Linear Static and Dynamic Finite Element Analysis*.

[31] Craig RR, Bampton MC (1968). Coupling of substructures for dynamic analyses. *AIAA Journal*.

[32] Craig RR, Chang CJ On the use of attachment modes in substructure coupling for dynamic analysis.

[33] Shabana AA (2010). *Computational Dynamics*.

[34] Ascher UM, Petzold LR (1998). *Computer Methods for Ordinary Differential Equations and Differential-Algebraic Equations*.

[35] Brenan KE, Campbell SL, Petzold LR (1996). *Numerical Solutions of Initial-Value Problems in Differential-Algebraic Equations*.

[36] García de Jalón J, Bayo E (1994). *Kinematic and Dynamic Simulation of Multibody Systems, the Real-Time Challenge*.

[37] Baumgarte J (1972). Stabilization of constraints and integrals of motion in dynamical systems. *Computer Methods in Applied Mechanics and Engineering*.

